# Human acid sphingomyelinase structures provide insight to molecular basis of Niemann–Pick disease

**DOI:** 10.1038/ncomms13082

**Published:** 2016-10-11

**Authors:** Yan-Feng Zhou, Matthew C. Metcalf, Scott C. Garman, Tim Edmunds, Huawei Qiu, Ronnie R. Wei

**Affiliations:** 1 Protein Engineering Department, Biologics Research, Sanofi, Framingham, Massachusetts 01701, USA; 2 Department of Biochemistry & Molecular Biology, University of Massachusetts Amherst, Amherst, Massachusetts 01003, USA

## Abstract

Acid sphingomyelinase (ASM) hydrolyzes sphingomyelin to ceramide and phosphocholine, essential components of myelin in neurons. Genetic alterations in ASM lead to ASM deficiency (ASMD) and have been linked to Niemann–Pick disease types A and B. Olipudase alfa, a recombinant form of human ASM, is being developed as enzyme replacement therapy to treat the non-neurological manifestations of ASMD. Here we present the human ASM holoenzyme and product bound structures encompassing all of the functional domains. The catalytic domain has a metallophosphatase fold, and two zinc ions and one reaction product phosphocholine are identified in a histidine-rich active site. The structures reveal the underlying catalytic mechanism, in which two zinc ions activate a water molecule for nucleophilic attack of the phosphodiester bond. Docking of sphingomyelin provides a model that allows insight into the selectivity of the enzyme and how the ASM domains collaborate to complete hydrolysis. Mapping of known mutations provides a basic understanding on correlations between enzyme dysfunction and phenotypes observed in ASMD patients.

## Abstract (web summary)

Genetic alterations in the protein acid sphingomyelinase (ASM) lead to ASM deficiency and have been associated with Niemann–Pick disease. Here, the authors report the crystal structures of ASM alone and bound to its product, and discuss the catalytic mechanism and its possible significance for patients with ASM deficiency.

## Graphical abstract

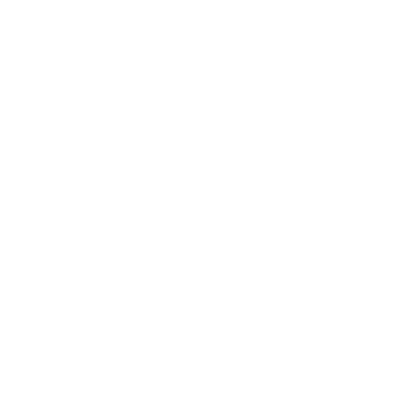

Acid sphingomyelinase deficiency (ASMD) causes abnormal accumulation of sphingomyelin in lysosomes, resulting in the progressive, life-threatening disorder historically referred to as Niemann–Pick disease types A (NPD A), presenting with an early-onset neuronopathic phenotype, and type B (NPD B), the non-neuronopathic form^1^. ASMD is a result of mutations in acid sphingomyelinase (ASM, E.C. 3.1.4.12), which hydrolyzes sphingomyelin to ceramide and phosphocholine (Fig. 1a)^2^. Recent studies on this rare disease illustrate that ASM plays an important role in the ceramide-mediated signalling pathway. Recombinant human ASM expressed from Chinese hamster ovary (CHO) cells is being developed as enzyme replacement therapy (ERT) for the non-neurological manifestations of ASMD, and is currently in the phase 1/2 pediatric trial and phase 2/3 trial for adults^3^,^4^.

ASM is highly conserved in animals, from *C. elegans* to human, with over 35% protein sequence identities (Supplementary Fig. 1). Sequence analysis suggests ASM is a multi-domain protein, including a saposin domain, a proline-rich linker, a metallo-dependent phosphatase catalytic domain, and an ill-defined C-terminal domain (Fig. 1b)^5^. Saposin proteins are sphingolipid activator proteins that present lipids from membranes to the active sites of various enzymes in an acidic cellular compartment^6^. The saposin domain in ASM is sufficient to support the hydrolysis of sphingomyelin without external saposin proteins^7^,^8^. In addition, ASM differs from neutral and alkaline sphingomyelinases in both domain architecture and protein sequence, with identities <10%.

Previous biochemical characterizations reveal that proper function of human ASM (UniProt database ID: P17405) requires zinc ions, multiple post-translational modifications, and acidic pH (ref. 5). On the basis of its localization, two forms of ASM originating from one gene have been reported, an intracellular lysosomal form and an extracellular secreted form^9^. Zinc ions are prerequisites for activity of both forms^10^. The lysosomal ASM is preloaded with zinc, while the secreted form requires exogenous zinc^9^. Also, human ASM has six potential *N*-linked glycosylation sites, and its *in vitro* activity pH optimum is around 5 (ref. 11). However, it is not clear how zinc and other physiological factors are involved in the enzyme function.

Despite its clinical significance, limited structural studies on ASM have been reported that elucidate its substrate selectivity and enzymatic mechanism. A model of human ASM catalytic domain has been generated based on a distant homolog, purple acid phosphatase^12^. Two recent crystal structures of catalytic domain of sphingomyelinase phosphodiesterase like 3a (SMPDL3A), sharing 31% identities to human ASM, are reported^13^,^14^. However, the understanding on ASMD is limited to the metal-binding site due to the low sequence identities and absence of the N-terminal saposin domain.

Here we report two crystal structures of recombinant human ASM and observe how co-factor zinc ions and a product phosphocholine are coordinated by the enzyme. The multi-domain structures, representing two states in the catalytic cycle, shed light on the mechanism of how natural substrate sphingomyelin is recognized and degraded. Moreover, currently reported ASMD mutations in the UniProt database were mapped onto the structure to build genotype–phenotype correlations with atomic details.

## Results

### Overall structure

Full-length human ASM was expressed from HEK293S *N*-acetylglucosaminyl transferase I-deficient (Gnt1^−^) cells with Man_5_ type glycans. ASM in cell culture media was purified with a monoclonal antibody coupled affinity column, followed by size exclusion column. Purified protein was crystallized at pH 5.5. The recombinant human ASM being developed for ERT, olipudase alfa, was also crystallized without any protease in the crystallization drops. Olipudase alfa was expressed in CHO cells with complex glycans^15^. The olipudase alfa crystals diffracted to around 4 Å at synchrotron (Table 1), and clear glycosylation and zinc cofactors densities were observed (Supplementary Fig. 2). Its refined structure was essentially identical to ASM from HEK cells. The descriptions in this report are focused on ASM from HEK cells unless specified.

**Figure 1:**
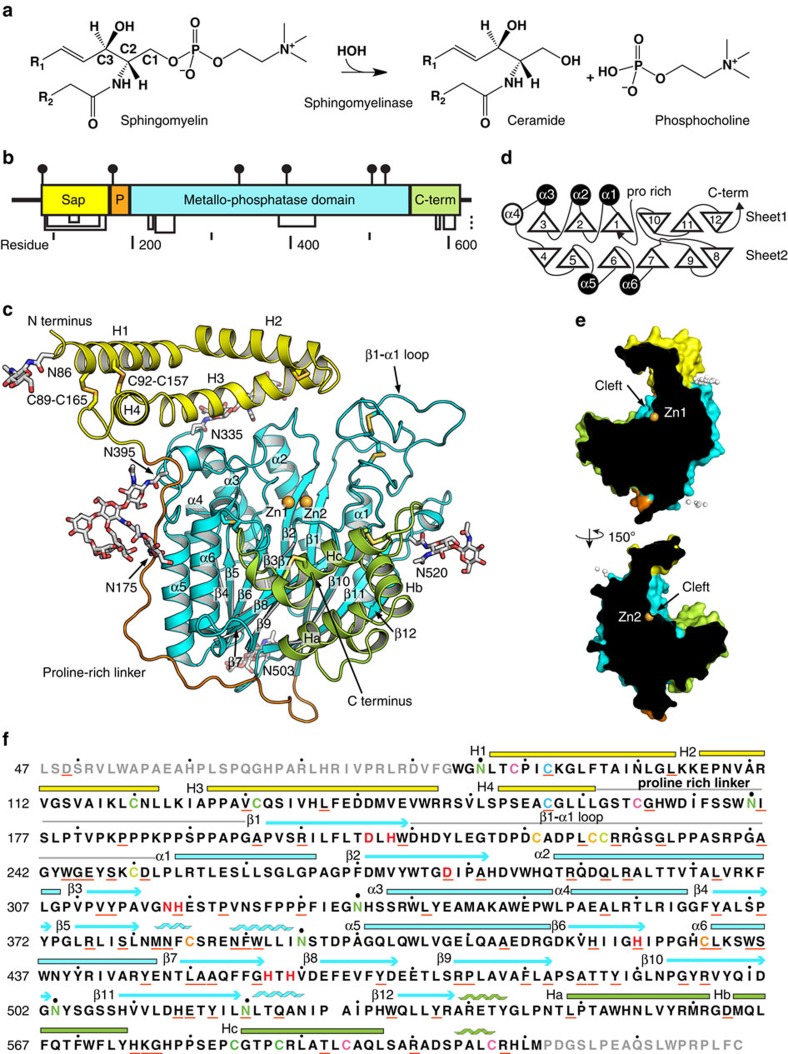
Structure of human ASM. (**a**) Sphingomyelin degradation catalyzed by ASM. (**b**) Diagram of ASM domains drawn to scale, with yellow saposin, orange proline-rich linker, and green C-terminal domain. (**c**) Overall structure of ASM. Glycosylation and disulfide bonds are in sticks. Two zinc ions are in spheres. (**d**) Topology of ASM. Upward and downward pointing triangles indicate the N to C terminus orientation of β strands. Open and closed circles indicate upward and downward going helices, respectively. (**e**) Cross-section illustration of cleft on ASM surface, with zinc and glycans as spheres. Arrow points to the center of substrate binding cleft. (**f**) ASM protein sequence with structural features. Disulfides are shown as matched cysteine colors. Residues that are not visible in the structure are grey. Underlined residues are reported ASMD mutation sites. Catalytic residues are bold and red. Glycosylated asparagine residues are green.

ASM shares <10% sequence identity to structurally known homologues. Therefore, the holoenzyme structure was determined using single-wavelength anomalous diffraction (SAD) from a Pt derivative at 2.43 Å, and the phases was extended to 2.25 Å using a Zn-SAD data set. The final model was refined to Rfree of 0.203 (Table 1). Clear electron density allowed us to build all residues from W84 to M611 as well as two zinc ions, six *N*-linked glycans, and eight disulfides (Fig. 1c). The same residue range is visible in the olipudase alfa structure, suggesting ASM N and C termini are inherently disordered and flexible. The structure includes an N-terminal saposin domain (residues 84–167), a proline-rich linker (168–195), a catalytic metallophosphatase domain (196–538), and a helical C-terminal domain (539–611) (Fig. 1).

### Saposin domain and proline-rich linker

The presence of saposin domain and proline-rich linker distinguishes ASM from all the other structurally known proteins. A number of crystal structures have demonstrated that saposin proteins undergo open and close conformational changes in response to lipid binding (Supplementary Fig. 3)^16^,^17^,^18^. The four helices in ASM saposin domain were observed in an extended open conformation in which helices H1 and H2 run nearly antiparallel to H3 and H4 (Fig. 1c). The helices are stabilized by two disulfide bonds connecting the saposin domain N and C termini and a third disulfide between H2 and H3. As a result, helices H3 and H4 form tight pairwise helical interactions with H1 and H2. The kinked H3 helix mediates saposin and catalytic domain interactions. In contrast, the H1 and H4 helices extend away from the catalytic domain, and there is no direct contact between H4 and catalytic domain (Fig. 1c). The four ASM saposin helices are amphipathic. The concave inner surface is hydrophobic and faces towards the zinc ions in active site, while the convex outer surface is lined with hydrophilic residues and exposed to solvent.

A 30-residue linker connects the saposin domain to the catalytic domain (Fig. 1c,f). The structure reveals that this linker spans 57 Å and covers 1128 Å^2^ surface of the catalytic domain. The linker is bent in the middle at position T180, resulting in a 97° kink. The linker can be divided to prolineless N-terminal half and proline-rich C-terminal half, based on amino-acid features. In the prolineless half, there is one glycosylation and several serine residues, but no proline. In contrast, the C-terminal half is dominated by proline, with one triple and two double proline motifs (P^182^KPPPKPPSPPAP). The prolineless half runs along the α5 and α6 in catalytic domain and is stabilized through extensive hydrophobic interactions with α5 and α6. The proline-rich half is extended and wraps around loops following α6, β8 and β10 in catalytic domain.

### Catalytic domain

The catalytic domain structure shows two layers of β-sheets, both six-stranded, form the central core, with dimensions 47 Å × 50 Å × 53 Å. Six α-helices and the helical C-terminal domain flank the β-sheets resulting in a four-layer α/β/β/α sandwich architecture. The catalytic domain starts at β1 in the center of the first sheet and ends at β12 at the edge of this sheet (Fig. 1d). The topological arrangement suggests catalytic domain adopts a metallophosphatase fold (SCOP d.159) and belongs to a calcineurin-like phosphoesterase superfamily in Pfam database (PF00149). ASM catalytic domain and C-terminal domain shares high structural similarity to SMPDL3A (refs 13, 14), with identical topology and root mean square deviation (r.m.s.d.) of 1.4 Å of all Cα atoms. Superimposition of ASM catalytic domain to pig purple acid phosphatase (PAP, PDB 1UTE) and bacteria neutral sphingomyelinase (PDB 2DDR) shows r.m.s.d. values of 3.1 and 3.9 Å, respectively. The unique topological arrangement of β9–10 and β10–12 strands makes the catalytic domains in ASM and SMPDL3A differ from currently identified members in metallophosphatase fold and neutral sphingomyelinases (DNase I-like fold, SCOP d.151)^19^.

**Figure 2:**
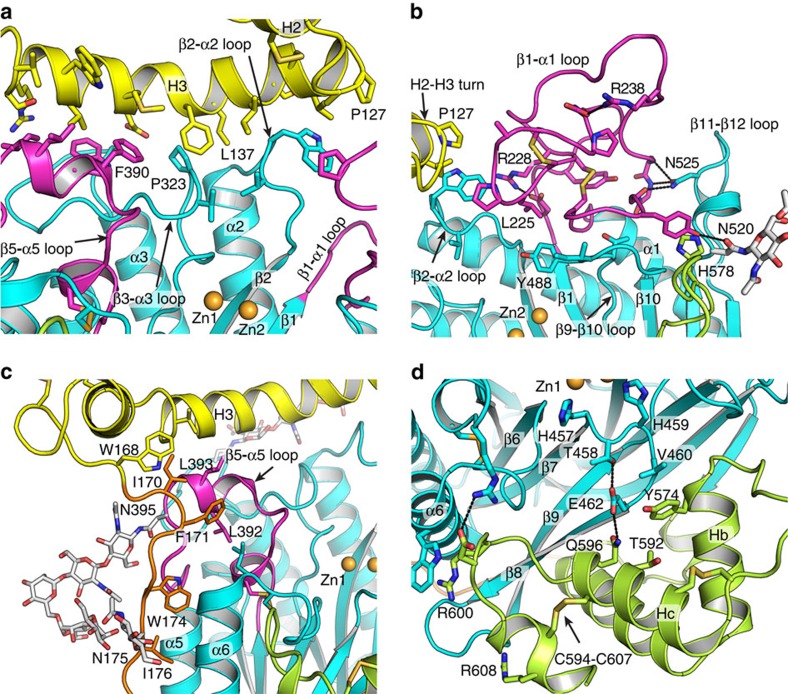
Structural details of the substrate binding cleft. (**a**) Interface between H3 in saposin and the β5-α5, β3-α3, β2-α2 loops in the catalytic domain. (**b**) β1-α1 loop in catalytic domain near by the tip of H2-H3 turn in saposin domain. (**c**) Hydrophobic interactions between N-terminal part of the proline-rich linker and catalytic domain. (**d**) Interface between the C-terminal domain and catalytic domain.

One feature of the ASM metallophosphatase domain is the long loops following certain β strands and extending toward the zinc ions (Fig. 2). These loops not only mediate zinc and substrate binding but also form the interfaces to the proline-rich linker, saposin and C-terminal domains. H3 helix in saposin domain forms extensive contacts to β1-α1, β2-α2, β3-α3 and β5-α5 loops (Fig. 2a,b). The interface between H3 and these loops is 700 Å^2^ and over 30 Å long. It is worth noting that many ASMD mutations occur on the interface residues, such as L137P in H3, P323A on the β3-α3 loop, ΔF390 and W391G on the β5-α5 loop. I170 and F171 on proline-rich linker interact with V150 on saposin H3 and L393 on β5-α5 loop, resulting in a turn towards the catalytic domain. The proline-rich linker is further stabilized by hydrophobic interactions between W174, I176, L178 and the α5 α6 helices (Fig. 2c). Moreover, the 44-residue β1-α1 loop extends out from the catalytic domain and folds into a small globular unit next to the H2-H3 turn in saposin domain (Fig. 2b). The conformation of this long loop is enforced by internal two pairs of charged interactions, two pairs of disulfides, and hydrophobic interactions. This loop is also interacting with β2-α2, β9-β10 and β11-β12 loops.

The catalytic domain shares a 1,632 Å^2^ interface with the C-terminal domain (Fig. 2d). The C-terminal domain starts after β12 in sheet1 and contains four helices, Ha-c, and a 3_10_ at the C terminus. The β8–9 strands at the edge of sheet2 deeply inserts into the space between these helices. Moreover, C-terminal domain contributes to the stabilization of the active site conformation, especially the β7–β8 loop. It has been reported that ΔT592 mutation causes severe ASMD in patients^20^. T592 is in the middle of Hc helix, which forms extensive interactions with the catalytic domain (Fig. 2d). Thus, we predict ΔT592 mutation has severe influence on ASM folding.

### Zinc binding in the active site

The long loops in the catalytic domain, together with saposin and C-terminal domains, create a deep and wide open cleft (Fig. 1c,e). The saposin and C-terminal domains are on the opposite sides of the catalytic domain. This is the only large and deep cleft (15 Å × 30 Å × 10 Å) on ASM. Six *N*-linked glycans scatter on the surface and all distribute far away from this cleft.

**Figure 3:**
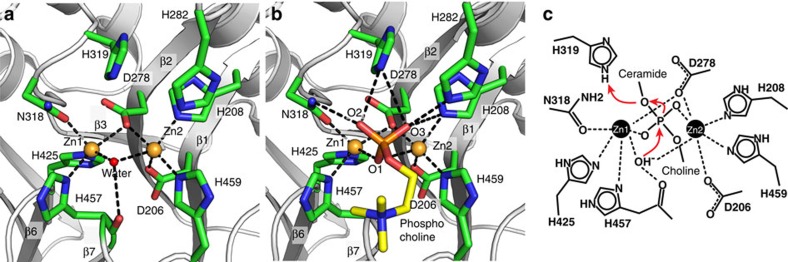
ASM active site. (**a**,**b**) Active sites of the holo (**a**) and phosphocholine bound (**b**) structures. Cα atoms are shown in grey ribbon. Two zinc atoms are shown as gold spheres, and one water molecule as red sphere. Phosphocholine is shown as sticks with yellow carbon, red oxygen and orange phosphorus. (**c**) Proposed catalytic mechanism in 2D diagram. Red arrows indicate electron relay during the cleavage. Only phosphate group in sphingomyelin are shown in details. Ceramide and choline indicate orientation of substrate in ASM.

Two neighbouring zinc ions are identified in the center of the cleft. The presence of zinc was confirmed by an X-ray fluorescence scan, and strong zinc anomalous signal has been observed in the data (Supplementary Fig. 4b). The location of zinc is at the C-terminal end of strands β1, β2, β6 and β7 (Figs 1c and 3a). The two zinc ions are separated by 3.5 Å and referred to as Zn1 and Zn2. Both zincs have trigonal bipyramidal geometry. D278 bridges between Zn1 and Zn2 at the axial positions, while H457 and H459 are in the other axial positions. Two of the equatorial positions are occupied by N318 and H425 for Zn1 and D206 and H208 for Zn2. Mutation of H425A or D206A abolishes ASM activity^12^, which is consistent with our structure. The conformations of the axial ligands H457 and H459 are constrained by side chains from helices in the C-terminal domain (Fig. 2d).

The precise side-chain arrangement around the metals facilitates recruitment of a key catalytic water molecule to the third equatorial position bridging between Zn1 and Zn2. The presence of this water is confirmed by observation on omit Fo-Fc map (Supplementary Fig. 4b). The catalytic water is stabilized and positioned by hydrogen bonds to the main chain carbonyl of H457 (Fig. 3a).There is an asymmetric arrangement in which the carboxylate oxygen of D278 is slightly closer to Zn2 (D-Zn1 2.5 Å, D-Zn2 1.9 Å), while the catalytic water is closer to Zn1 (Wat-Zn1 1.9 Å, Wat-Zn2 2.6 Å). In contrast, the coordination of this water in PAP and SMPDL3A is more symmetric (Supplementary Fig. 5)^14^.

### Phosphocholine binding

To better understand the ASM catalytic mechanism and how the Zn ions mediate hydrolysis, we soaked a product phosphocholine into ASM crystal and solved the structure to 2.5 Å (Table 1). Clear density was observed near the zinc atoms in the Fo-Fc difference map (Supplementary Fig. 4c). One phosphocholine was built into the electron density, with the choline pointing toward the C-terminal domain (Fig. 3b). The phosphoryl group is tightly coordinated by both zinc ions and the neighbouring catalytic residues. Oxygen (O1) on the phosphoryl group replaces the position of the catalytic water molecule in the holo ASM structure. N318 and H319 on β3-α3 loop, H208 on β1, and H282 on β2-α2 loop interact with two oxygen atoms (O2 and O3) on the phosphoryl group.

## Discussion

Sphingomyelin is a key membrane component from bacteria to human. Understanding of how sphingomyelin is recognized and hydrolysed by ASM greatly enhances our knowledge of sphingolipid metabolism and associated human diseases. Due to low sequence identity, reliable prediction of human ASM function cannot be made based on structurally known proteins. The human ASM structures we report here illustrate how the co-factor zinc ions activate the enzyme and how domain arrangement contributes to substrate specificity.

A putative catalytic mechanism can be deduced for ASM, based on the structures (Fig. 3c). We crystallized two states in the ASM catalytic cycle, the holoenzyme state, and product phosphocholine bound state. We propose that ASM catalysis is an associative general acid–base type reaction.

**Figure 4:**
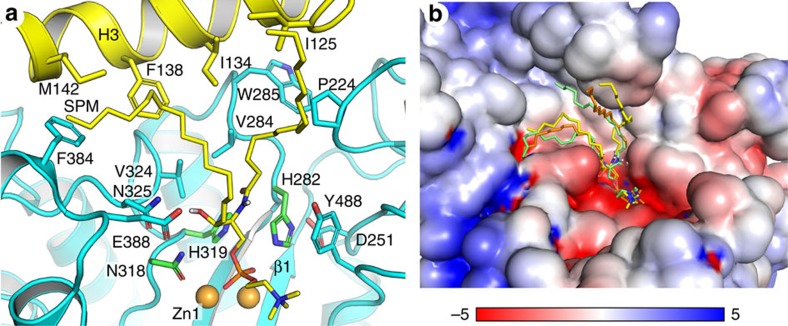
Docked model of sphingomyelin on ASM. (**a**) Surface residues around C-16 sphingomyelin. Zinc atoms are shown gold spheres. Sphingomyelin is coloured as yellow carbon, blue nitrogen, red oxygen and orange phosphorus. (**b**) Electrostatic potential surface calculated at pH 5.0 with *PropKa* (ref. 46) and *PDB2PQR* (ref. 47). Red and blue surfaces correspond to negative and positive electrostatic potential scaled from −5 *k*_*b*_*T* to 5 *k*_*b*_*T*. Three docked poses were shown on the surface, and the yellow pose was selected and shown in **a**.

The active site in ASM defines a stringent specificity for zinc and the phosphate group of sphingomyelin. The two zinc ions are coordinated by four histidines, two aspartic acids, one asparagine and the catalytic water molecule. During the substrate recognition, we speculate that two zinc co-factors serve as anchors for the two phosphate oxygens from sphingomyelin. The water can be deprotonated to hydroxide, because the two close zinc ions extract proton away from this water. Following that, the hydroxide initiates nucleophilic attack on the phosphorus of sphingomyelin. The arrangement of the active site allows the phosphate oxygen on the ceramide side to extra a proton from either H319 or H282 (Fig. 4a). The H282 imidazole ring is stabilized by *π*–*π* stacking interaction with Y488 and polar interaction to D251 carboxylate group, while the H319 is involved in water mediated hydrogen bonding. As a result, H319 is a more energy favoured hydrogen donor in the reaction. We propose the imidazole ring on H319 donates a proton to the oxyanion of the ceramide leaving group. It is worth noting that the cleft is open and solvent accessible, which allows quick exchange of protons on both H319 and the nucleophile water in the regeneration step. Consistent with this hypothesis, mutation of H319 to tyrosine causes severe ASMD^21^.

The proposed mechanism is further supported by observations in other di-metal-binding metallophosphatases. Despite <10% protein sequence identities, ASM shares a conserved catalytic site and di-metal binding pattern with other metallophosphatases, such as mammalian purple acid phosphatase (Supplementary Fig. 5)^22^. The di-metal bridging water molecule is found at the active site in this class of catalytic domains/enzymes. In addition, the phosphate location is also conserved, with the same trigonal bipyramidal coordination geometry.

Most of the sphingolipid degradation enzymes require activator proteins, like saposins, to present substrates to the catalytic domain. ASM has its own saposin domain for sphingomyelin recognition. Our structure provides direct observation on how saposin and catalytic domain are arranged for sphingomyelin recognition.

Major sphingomyelin species in biological membranes have long acyl chains, with three dominant lengths, C-16, C-18, and C-24 (ref. 23). The substrate binding cleft in ASM is created by the catalytic domain in the middle, and saposin domain and C-terminal domain at two opposite sides. Zinc ions and phosphocholine occupy the center and the C-terminal domain side of the cleft. The saposin concave surface facing towards the active site is rich in hydrophobic residues (Fig. 4a) and not highly charged (Fig. 4b), which can accommodate the hydrophobic ceramide chains in sphingomyelin. The distance between Zn1 and the phenyl ring of F138 in H3 is 14 Å, which is close to the 18 Å length of an extended 16 carbon chain Ahn *et al*.^16^ have shown that hydrophobic chains of a phosphatidylethanolamine are accommodated within a hydrophobic cavity at the middle of the kinked H3 helices in saposin B protein (Supplementary Fig. 3c). Recent crystal structure of saposin A lipoprotein discs and cryoelectron microscopic study show sapsoin proteins can wrap around lipids and membrand protein and form lipoprotein-like nanoparticles^18^,^24^. These observations indicate saposin might be functional as long as it is under open conformation, regardless its oligomeric states. With limited knowledge of binding between saposin proteins and lipids, we did docking of C-16 sphingomyelin onto ASM to visualize how the ceramide chains can be placed between the active site and the saposin domain (Fig. 4). The saposin is under open conformation in our structures. Even with the constraints of phosphocholine and the kinked region in H3 as docking site, the flexible ceramide chains were placed various positions (Fig. 4b), instead of form a specific interaction network with saposin domain. The modelling result suggests that sphingomyelins can fit well into the cleft on ASM surface. We propose it undergoes close–open conformational changes in response to substrate binding like other saposin proteins. The proline-rich linker could act as a hinge that switches the saposin domain conformational changes, facilitating presentation of substrate to the catalytic domain.

The chemical composition of the hydrocarbon chains allows sphingomyelin to fit better into ASM cleft than other phospholipids. Oninla *et al*.^25^ has found that ASM catalyses degradation of C18-sphingomyelin 10-fold faster than dipalmitoyl-phosphatidylcholine, although they share an identical phosphocholine head group. Sphingomyelin in nature is synthesized with two stereo chemical requirements in the hydrocarbon chain: the double bond in sphingosine is in *trans* configuration, and the C2 amine and C3 hydroxyl must have threo relationship^26^. We speculate that these are the two key factors for proper substrate alignment on ASM. First, the amine at C2 and hydroxyl at C3 positions are good hydrogen-bond donors (Fig. 1a), while the glycerol backbone in phosphatidylcholine has fewer hydrogen-bond donors. N325, E388 and Y488 in ASM are within 7 Å from the zinc ions and in position for hydrogen bonding with substrate (Fig. 4a), and N325 is highly conserved among different species (Supplementary Fig. 1). Hydrogen bonding plus charged interactions with zinc ions together make substrate recognition highly specific. Second, naturally existing sphingomyelin is much more saturated than phosphatidylcholine, which normally contains one or more double bonds in *cis* configuration in the middle of the acyl chains^23^. The rigidity of the *cis* double bond causes the acyl chain to bend and restricts the conformational freedom. As a result, we predict the recognition and presentation of phosphatidylcholine and other phospholipids to ASM active site is slower than that of sphingomyelin.

**Figure 5:**
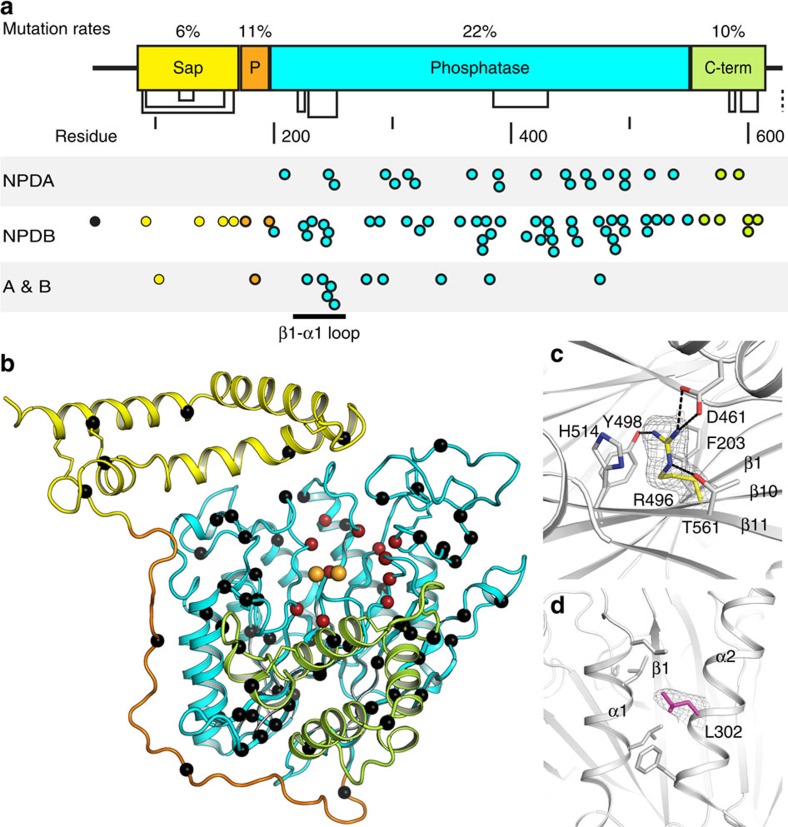
Mutations mapped on structure. (**a**) Distribution of mutations on human ASM 1-D sequence. Each point represents one reported mutation in the UniProt database. Points are coloured according to domains. (**b**) Distribution of mutations on 3D structure. Cα atoms of mutated residues are shown as spheres. Activity-related mutations are red, and folding related ones are black. Zinc atoms are gold spheres. (**c**) Network of R496 with neighbouring residues in the hydrophobic core between β-sheets. (**d**) Hydrophobic interactions mediated by L302 between α1 and α2 helices. In **c**–**d**, 2Fo-Fc density for side chains of mutation sites are shown and contoured at 1*σ*.

ASMD is caused by loss of function mutations in ASM. There are ninety known mutations listed in UniProt database and summarized in supplementary Table 1. All mutations are covered in our structure, except D49V located in the signal peptide. Mapping mutations onto one dimensional (1D) protein sequence and 3D structure reveals interesting occurrence pattern in human ASM (Fig. 5). Overall, 82% of the mutations are located in the catalytic domain, resulting in 21.6% mutation rate in protein sequence. In contrast, the mutation rates in the saposin, proline-rich linker, and C-terminal domains are no >11% (Fig. 5a).

Those mutations are predicted into two categories according to their disruptive effects on catalytic activity and protein folding (Fig. 5b; Supplementary Table 1). First, the catalytic group includes mutation of residues that are directly or indirectly involved in zinc binding or phosphocholine binding in the specificity pocket. The indirect residues disrupt the folding near the active site and thus affect catalysis. For direct coordination, D278A mutation eliminates the coordination to both zinc atoms. H319Y not only introduces a larger group to the compact active site but also loses the imidazole group which plays an essential role in the phosphodiester bond cleavage. H425 points the imidazole ring towards zinc atoms, and mutation to the larger arginine completely disrupts zinc coordination. There are many other mutations that affect the active site indirectly. For example, the A281T mutation affects H319, and W209R mutation disrupts the β1 stability and affects D206 and H208, which coordinate Zn2. Q292 and H319 are involved in a buried hydrogen-bond network including A281 and Q287. The Q292 amide forms a 3.2 Å hydrogen bond with the H319 carbonyl. The Q292K mutation brings a longer side chain into this well-defined hydrogen bonding network and perturbs the catalytic role of H319.

Among the mutations in the catalytic domain, there are two clusters in the 3D structure. One is on the tightly folded β1-α1 loop (Fig. 2b). Mutations like A241V, G242R, G245S, introduce larger side chains and disrupt local folding. The other cluster spreads onto β4, β5, β5-α5 loop and β6-α6 loop in the protein sequence. According to the structure, these mutations are in close proximity to H3 in the saposin domain, a critical location for binding of sphingomyelin.

The second group of mutations is on residues that are widely distributed in the structure and distant from the active site. These mutations destabilize local interaction networks and affect enzyme stability and folding (Fig. 5b). Most of the residues are sequestered from solvent (Supplementary Table 1), and some surface exposed ones are heavily involved in interactions with neighboring residues in the folded structure. R496L and L302P mutations are two of the three prevalent mutations in the Jewish population and cause NPD A. Both R496 and L302 are highly conserved among species (Supplementary Fig. 1). The guanidinium group of R496 forms extensive interactions with neighboring residues, and stabilize the buried core between the β-sheets (Fig. 5c). L302 is located in the middle of helix α2, and the local hydrophobic interactions maintain the stability of α1 and α2, which are next to β1 strand in the active site (Fig. 5d). In addition, mutation to a proline, which often interrupts helical structures, further decreases the local stability.

Deletion of R608 (ΔR608) is the most common mutation in NPD B, and patients with homozygous ΔR608 mutations have mild phenotype. R608 is located after the C594-C607 disulfide bond on a 3_10_ helix. Residues after M611 are missing electron density and presumably disordered. The residues after R608 show very low sequence identities among ASM from different species (Supplementary Fig. 1), but a free cysteine at the C-terminal end is conserved and the length of this region in most species is around 20 amino acids. Mutation or deletion of the last residue C629 in human ASM is known to increase ASM activity and the cysteine is proposed to form direct contacts to active site^27^. It has been reported in other metallophosphatase, like PhoD in Bacillus subtilis, that the C-terminal end folds back to active site to inhibit enzyme activity^28^. We predict that deletion of R608 will disturb the C terminus and affect its activity. In contrast to ΔR608, ΔT592 has been reported as an NPD A mutation. As described earlier, T592 forms direct contacts with active site. ΔT592 will disrupt the interactions between the C-terminal domain and the catalytic domain, resulting in a perturbed active site and severe enzyme deficiency (Fig. 2d).

Taken together, we predict how individual mutation affect enzyme folding and function based on the atomic resolution structure of ASM. This information allows us to better understand gene mutations and ASMD phenotypes in the context of enzyme folding and catalytic activity. As abovementioned, we observe consistent correlations between disease severity and a disruptive effect on the ASM active site or folding. The structural information could serve as a guide to analyse and predict the phenotypic outcome in ASMD individuals. The structures provide us the ability to understand the molecular mechanism behind diseases. Following tests of theromostability and enzymatic assay with ASM mutants will help us better reveal the predicted correlations.

The abovementioned structures open a door for us to decipher ASM catalytic mechanism and mutations in ASMD patients. However, many questions remain unanswered. First one will be how ASM interacts with membrane and reaches sphingomyelin. ASM in solution is monomeric, and it has not been reported that ASM needs to dimerize to function. New evidence with different techniques may better reveal the details. Another question is how ASM is inhibited by the anti-depressants drugs. Model has been proposed that those tricyclic drugs would insert into membrane and adjust charges on the surface, which will interfere the electrostatic adherence of ASM to membrane^29^. The structure shown here does not conflict with this model. However, these hydrophobic tricyclic drugs could also potentially bind to the hydrophobic surface in sapson domain thus prevent saposin–sphingomyelin interaction. A following co-crystal structure of ASM or saposin domain with these small molecules would provide more insights. Moreover, ASM activity is regulated by different modifications and physiological partners, such as phosphorylation on S508 and association with acid ceramidase^30^,^31^. We observe S508 on β10–β11 loop is partially buried by the proline-rich linker and hydroxyl group is hydrogen bonded to D501 in our structures. This serine residue is not phosphorylated and located in a position distant from substrate binding site. A crystal structure with phosphorylated S508 may address the question. ASM could also be activated by redox conditions and possible through the last free cysteine C629 (refs 27, 32). We see that most of the disulfides in ASM are solvent accessible and the C-terminal C629 are exposed and too flexible to be seen in our structures. The exposure of these residues may make ASM sensitive to redox conditions.

We present the atomic resolution structures of human ASM here, with detailed information about zinc and phosphocholine binding. The structures allow us to interpret zinc activation and substrate hydrolysis in ASM. In addition, the results provide insights about how catalytic domain and its neighboring saposin, proline-rich linker, and C-terminal domains together create a cleft specific for sphingomyelin binding. These data also give us the opportunity to start correlating mutations in patients and enzyme deficiency.

## Methods

### Protein expression

Codon optimized DNA encoding human ASM (M1 to C629) was cloned into the NheI and BamHI sites of plasmid pIRES2-EGFP and confirmed by sequencing. HEK293S Gnt1^−^ cells (ATCC CRL-3022) were transfected using polyethylenimine^33^. Single colonies were selected in Dulbecco's modified Eagle's medium and 5% fetal bovine serum with 1 mg ml^−1^ G418. Stably transfected cells were cultured in suspension in Freestyle 293 serum-free medium, and supernatants were harvested 7 days after expansion.

Olipudase alfa was produced at Sanofi Genzyme^27^. Human full-length ASM cDNA was cloned into a dihydrofolate reductase selection vector and stably expressed in CHO-DXB11 cells (ATCC CRL-9096).

### Protein purification

HEK293S Gnt1^−^ cell expressed ASM was purified from the supernatant using antibody affinity and gel-filtration chromatography. Anti-ASM monoclonal antibody was coupled to AminoLink Plus resin (Thermo Scientific, USA) following the manufacturer's protocol. ASM supernatant was adjusted to 20 mM Bis-Tris pH 6.0, 0.15 M sodium chloride and 0.1 mM zinc acetate, and incubated with resin overnight at 4 °C. ASM was eluted with 20 mM sodium citrate pH 3.0, and neutralized with 0.1 M Bis-Tris pH 6.5. The eluate was further purified using Superdex 200 10/300 GL (GE healthcare, USA) in 20 mM Bis-Tris pH 6.0, 0.15 M sodium chloride (Supplementary Fig. 6). Peak fractions were concentrated to 20 mg ml^−1^.

Olipudase alfa was purified from culture media using hydrophobic interaction chromatography followed by ion exchange chromatography. Eluate was concentrated and subjected to gel-filtration as described in purification of ASM from HEK293S Gnt1^−^ cells. The monomer peak was eluted 0.9 ml earlier than ASM from HEK293 S Gnt1^−^ cells (Supplementary Fig. 6). Peak fractions were collected and concentrated to 20 mg ml^−1^ for crystallization.

### Crystallization

ASM was crystallized with *in situ* proteolysis of endoproteinase Glu-C at 1:200 protease:ASM mass ratio. Crystals were obtained in sitting drops at 21 °C using a well solution of 1.5 M ammonium sulfate, 0.1 M sodium acetate pH 5.0–5.5, 12 % glycerol. Olipudase alfa crystals were obtained at 21 °C in the same condition, but without any protease in the drops.

Platinum derivatives were prepared by soaking native crystals in well solution supplemented with 10 mM K_2_PtCl_4_ for 10 min at 21 °C. Derivatized crystals were back soaked for 5 min in a cryo protectant solution supplemented with 20% glycerol and flash frozen in liquid nitrogen.

The phosphocholine bound crystals were generated by soaking native crystals in well solution supplemented with 30 mM phosphocholine for 15 min at 21 °C. Crystals were collected as with Pt-derivatives.

### Data collection

X-ray fluorescence scans identified zinc in crystals. SAD data were collected at beamline SER-CAT 22ID at the Advanced Photon Source (APS, USA). Diffraction from native crystals was collected at the Zn peak wavelength (1.2830 Å) and Pt-derivatized crystals at the Pt L-III edge (1.0721 Å). Phosphocholine-soaked data were collected on a Rigaku FR-E+ X-ray generator with a Cu anode and Saturn 944+ CCD detector. Zn and Pt SAD data were processed with HKL2000 (ref. 34), and phosphocholine data were processed with iMosflm and Scala^35^,^36^. Diffraction of olipudase alfa was performed at the Zn peak wavelength (1.2825 Å) at beamline CMCF-08ID at the Canadian light source, and the SAD data were processed with XDS^37^,^38^. Data collection statistics are listed in Table 1.

### Structure determination

Pt-SAD data were used in *phenix.autosol* for Pt location search, phasing and density modification^39^. Six Pt sites were found (Supplementary Fig. 4a), and the figure of merit for phasing was 0.251. The density-modified map was loaded into *buccaneer* in CCP4 for protein-chain building^40^. Over 90% of the ASM residues were traced.

The holo ASM structure was completed using the higher-resolution Zn-SAD data. The output model from Pt-SAD was put into *phaser* MR-SAD^41^ against the Zn-SAD data for Zn searching and phasing. Two zinc atoms were located in each protein monomer (Supplementary Fig. 4b). The model was refined against anomalous amplitudes with *phenix.refine*^42^, and Hendrickson–Lattman coefficients from *phaser* MR-SAD were applied during refinement. Model building was completed with Coot^43^. The sequence-to-structure register was guided by the glycosylation sites and disulfide bonds. Continuous electron density was observed from W84 to M611, and all main chain atoms are visible in the electron density.

The holo structure was used as a search model for MR phasing of phosphocholine data with *phaser*. One phosphocholine was built into the Fo-Fc difference map with *Coot* (Supplementary Fig. 4c), and refinement was completed with *phenix.refine*.

Structure determination and refinement of olipudase alfa were the same as described for holo ASM. Three protein monomers were found in each asymmetric unit. NCS restraints were applied, and high resolution ASM holoenzyme structure was used as reference structure during the refinement with *phenix.refine*. Two zinc atoms were located in each monomer according to the anomalous difference map (Supplementary Fig. 2b). Glycosylations on N175, N335, N503 and N520 were clearly visible in omit Fo-Fc maps (Supplementary Fig. 2a). Residues W84 to M611 were built into the electron density.

The final statistics for model building and refinement are listed in Table 1. Figures of structures are generated with *PyMOL*^44^.

### Docking sphingomyelin to ASM

To better visualize how the hydrocarbon chains might be placed on ASM upon substrate binding, a carbon-16 (C-16) sphingomyelin was docked to holo ASM with MOE 2014 (ref. 45). Amber10: EHT force field and R-field solvation were used. The ASM structure was prepared for 3D protonation with default parameters except that histidine, asparagine and glutamine side-chain conformations were retained. Sphingomyelin and ASM were energy minimized with default parameters. Phosphocholine interacting residues and zinc ions were selected as dock sites. In addition, I134, F138, and M142 in the saposin H3 helix were also included as dock sites based on the published saposin B and lipid crystal structure^16^. Phosphocholine was selected as ligand template, and template similarity placement protocol and induced fit protocol were applied in the docking procedure. Duplicated poses were removed and 30 energy favoured poses were retained after two rounds of rescoring. Other parameters were set as default. This docking is limited by the lack of computational restraints on two nearby zinc ions and numerous rotatable bonds in sphingomyelin. The wide open substrate binding cleft also contributed to the various conformations of hydrocarbon chains in docked results. The output 30 poses of sphingomyelin were visually inspected and validated based on whether the pose made reasonable interactions with ASM, instead of only looking for the best energy favored position. Three poses were selected and shown in Fig. 4.

### Data availability

Coordinates and structure factors have been deposited in the Protein Data Bank under accession code 5I81 (holo-ASM), 5I85 (ASM with phosphocholine) and 5I8R (olipudase alfa).

## Additional information

**How to cite this article**: Zhou, Y.-F. *et al*. Human acid sphingomyelinase structures provide insight to molecular basis of Niemann–Pick disease. *Nat. Commun.* **7**, 13082 doi: 10.1038/ncomms13082 (2016).

## Supplementary Material

Supplementary Information Supplementary Figures 1-6, Supplementary Table 1 and Supplementary References

## Figures and Tables

**Table 1:** Data collection and refinement statistics.

**Data collection**	**Holo ASM**	**Pt derivative**	**Phosphocholine**	**Olipudase alfa**
X-ray wavelength (Å)	1.2830	1.0721	1.5418	1.2825
Resolution (Å)	43.81–2.25 (2.33–2.25)	42.33–2.43 (2.52–2.43)	31.18–2.50 (2.64–2.50)	45.00–3.65 (3.74–3.65)
Space group	P6_4_22	P6_4_22	P6_4_22	I222
No. of reflections	88030	68691	33985	118574
*Cell dimensions*				
a, b, c (Å)	132.5, 132.5, 189.8	132.2, 132.2, 189.3	131.6, 131.6, 188.6	191.0, 230.9, 252.3
α, β, γ (°)	90.0, 90.0, 120.0	90.0, 90.0, 120.0	90.0, 90.0, 120.0	90.0, 90.0, 90.0
*I*/*σ*(*I*)	22.9 (1.9)	22.1 (2.0)	12.6 (3.2)	7.44 (0.34)
Rmerge	0.092 (0.732)	0.081 (0.735)	0.200 (0.822)	0.229 (5.35)
Completeness (%)	99.5 (94.9)	99.0 (90.9)	99.9 (100.0)	98.9 (86.3)
Redundancy	10.3 (5.0)	7.3 (4.9)	17.3 (13.9)	6.9 (5.4)
CC1/2 in highest shell	0.669	0.743	0.852	0.108
*Phasing and refinement*				
Phasing figure of merit	—	0.251	—	—
*R*_work_/*R*_free_	0.184/0.203		0.190/0.222	0.248/0.254
Monomers per asymmetric unit	1	1	1	3
Composition per asymmetric unit				
Amino acid/sugar	527/14		527/13	1581/29
Water/SO_4_	177/11		261/11	0//0
Zn/Phosphocholine	2/0		2/1	6/0
rmsd				
Bond (Å)	0.009		0.018	0.013
Angle (°)	0.902		0.885	0.931
Ramachandran plot (favoured/allowed/outlier)	96.4/3.6/0.0		95.4/4.6/0.0	95.6/4.2/0.2

ASM, acid sphingomyelinase; rmsd, root mean square deviation.

Holo ASM and Pt derivative are anomalous data collected at Zn peak and Pt L-III edge, respectively. Phosphocholine data set is non-anomalous. Olipudase Alfa data set is collected at Zn peak. Values in parentheses are for the highest-resolution shell.

## References

[b1] Schuchman E. H. & Desnick R. J. in The Online Metabolic and Molecular Bases of Inherited Disease eds Beaudet A. L.. The McGraw-Hill Companies, Inc. (2013).

[b2] Besley G. T., Hoogeboom A. J., Hoogeveen A., Kleijer W. J. & Galjaard H. Somatic cell hybridisation studies showing different gene mutations in Niemann-Pick variants. Hum. Genet. 54, 409–412 (1980). 6249719 10.1007/BF00291589

[b3] McGovern M. M. . Novel first-dose adverse drug reactions during a phase I trial of olipudase alfa (recombinant human acid sphingomyelinase) in adults with Niemann-Pick disease type B (acid sphingomyelinase deficiency). Genet. Med. 18, 34–40 (2016). 25834946 10.1038/gim.2015.24

[b4] Wasserstein M. P. . Successful within-patient dose escalation of olipudase alfa in acid sphingomyelinase deficiency. Mol. Genet. Metab. 116, 88–97 (2015). 26049896 10.1016/j.ymgme.2015.05.013 PMC4561589

[b5] Jenkins R. W., Canals D. & Hannun Y. A. Roles and regulation of secretory and lysosomal acid sphingomyelinase. Cell Signal. 21, 836–846 (2009). 19385042 10.1016/j.cellsig.2009.01.026 PMC3488588

[b6] Kolter T. & Sandhoff K. Principles of lysosomal membrane digestion: stimulation of sphingolipid degradation by sphingolipid activator proteins and anionic lysosomal lipids. Annu. Rev. Cell Dev. Biol. 21, 81–103 (2005). 16212488 10.1146/annurev.cellbio.21.122303.120013

[b7] Paton B. C., Schmid B., Kustermann-Kuhn B., Poulos A. & Harzer K. Additional biochemical findings in a patient and fetal sibling with a genetic defect in the sphingolipid activator protein (SAP) precursor, prosaposin. Evidence for a deficiency in SAP-1 and for a normal lysosomal neuraminidase. Biochem J 285, (Pt 2): 481–488 (1992). 1637339 10.1042/bj2850481 PMC1132813

[b8] Bradova V. . Prosaposin deficiency: further characterization of the sphingolipid activator protein-deficient sibs. Multiple glycolipid elevations (including lactosylceramidosis), partial enzyme deficiencies and ultrastructure of the skin in this generalized sphingolipid storage disease. Hum. Genet. 92, 143–152 (1993). 8370580 10.1007/BF00219682

[b9] Schissel S. L., Schuchman E. H., Williams K. J. & Tabas I. Zn^2+^-stimulated sphingomyelinase is secreted by many cell types and is a product of the acid sphingomyelinase gene. J. Biol. Chem. 271, 18431–18436 (1996). 8702487 10.1074/jbc.271.31.18431

[b10] Schissel S. L., Keesler G. A., Schuchman E. H., Williams K. J. & Tabas I. The cellular trafficking and zinc dependence of secretory and lysosomal sphingomyelinase, two products of the acid sphingomyelinase gene. J. Biol. Chem. 273, 18250–18259 (1998). 9660788 10.1074/jbc.273.29.18250

[b11] Callahan J. W., Jones C. S., Davidson D. J. & Shankaran P. The active site of lysosomal sphingomyelinase: evidence for the involvement of hydrophobic and ionic groups. J. Neurosci. Res. 10, 151–163 (1983). 6313952 10.1002/jnr.490100205

[b12] Seto M. . A model of the acid sphingomyelinase phosphoesterase domain based on its remote structural homolog purple acid phosphatase. Protein Sci. 13, 3172–3186 (2004). 15557261 10.1110/ps.04966204 PMC2287300

[b13] Lim S. M., Yeung K., Tresaugues L., Ling T. H. & Nordlund P. The structure and catalytic mechanism of Human Sphingomyelin Phosphodiesterase like 3a - an acid sphingomyelinase homolog with a novel nucleotide hydrolase activity. FEBS J. 1107–1123 (2016). 26783088 10.1111/febs.13655

[b14] Gorelik A., Illes K., Superti-Furga G. & Nagar B. Structural basis for nucleotide hydrolysis by the acid sphingomyelinase-like phosphodiesterase SMPDL3A. J. Biol. Chem. 291, 6376–6385 (2016). 26792860 10.1074/jbc.M115.711085 PMC4813564

[b15] Stanley P., Schachter H. & Taniguchi N. in Essentials of GLycobiology eds Varki A., Cummings R. D., Esko J. D. Cold Spring Harbor Laboratory Press (2009). 20301239

[b16] Ahn V. E., Faull K. F., Whitelegge J. P., Fluharty A. L. & Prive G. G. Crystal structure of saposin B reveals a dimeric shell for lipid binding. Proc. Natl Acad. Sci. USA 100, 38–43 (2003). 12518053 10.1073/pnas.0136947100 PMC140876

[b17] Ahn V. E., Leyko P., Alattia J. R., Chen L. & Prive G. G. Crystal structures of saposins A and C. Protein Sci. 15, 1849–1857 (2006). 16823039 10.1110/ps.062256606 PMC2242594

[b18] Popovic K., Holyoake J., Pomes R. & Prive G. G. Structure of saposin A lipoprotein discs. Proc. Natl Acad. Sci. USA 109, 2908–2912 (2012). 22308394 10.1073/pnas.1115743109 PMC3286916

[b19] Openshaw A. E., Race P. R., Monzo H. J., Vazquez-Boland J. A. & Banfield M. J. Crystal structure of SmcL, a bacterial neutral sphingomyelinase C from Listeria. J. Biol. Chem. 280, 35011–35017 (2005). 16093240 10.1074/jbc.M506800200

[b20] Rodriguez-Pascau L. . Identification and characterization of SMPD1 mutations causing Niemann-Pick types A and B in Spanish patients. Hum. Mutat. 30, 1117–1122 (2009). 19405096 10.1002/humu.21018 PMC2760245

[b21] Sikora J., Pavlu-Pereira H., Elleder M., Roelofs H. & Wevers R. A. Seven novel acid sphingomyelinase gene mutations in Niemann-Pick type A and B patients. Ann. Hum. Genet. 67, 63–70 (2003). 12556236 10.1046/j.1469-1809.2003.00009.x

[b22] Guddat L. W. . Crystal structure of mammalian purple acid phosphatase. Structure 7, 757–767 (1999). 10425678 10.1016/s0969-2126(99)80100-2

[b23] Barenholz Y. & Thompson T. E. Sphingomyelin: biophysical aspects. Chem. Phys. Lipids 102, 29–34 (1999). 11001558 10.1016/s0009-3084(99)00072-9

[b24] Frauenfeld J. . A saposin-lipoprotein nanoparticle system for membrane proteins. Nat. Methods 13, 345–351 (2016). 26950744 10.1038/nmeth.3801 PMC4894539

[b25] Oninla V. O., Breiden B., Babalola J. O. & Sandhoff K. Acid sphingomyelinase activity is regulated by membrane lipids and facilitates cholesterol transfer by NPC2. J. Lipid Res. 55, 2606–2619 (2014). 25339683 10.1194/jlr.M054528 PMC4242453

[b26] Sribney M. & Kennedy E. P. The enzymatic synthesis of sphingomyelin. J. Biol. Chem. 233, 1315–1322 (1958). 13610834

[b27] Qiu H. . Activation of human acid sphingomyelinase through modification or deletion of C-terminal cysteine. J. Biol. Chem. 278, 32744–32752 (2003). 12801930 10.1074/jbc.M303022200

[b28] Rodriguez F. . Crystal structure of the Bacillus subtilis phosphodiesterase PhoD reveals an iron and calcium-containing active site. J. Biol. Chem. 289, 30889–30899 (2014). 25217636 10.1074/jbc.M114.604892 PMC4223295

[b29] Beckmann N., Sharma D., Gulbins E., Becker K. A. & Edelmann B. Inhibition of acid sphingomyelinase by tricyclic antidepressants and analogons. Front. Physiol. 5, 331 (2014). 25228885 10.3389/fphys.2014.00331 PMC4151525

[b30] Zeidan Y. H. & Hannun Y. A. Activation of acid sphingomyelinase by protein kinase Cdelta-mediated phosphorylation. J. Biol. Chem. 282, 11549–11561 (2007). 17303575 10.1074/jbc.M609424200

[b31] He X. . Purification and characterization of recombinant, human acid ceramidase. Catalytic reactions and interactions with acid sphingomyelinase. J. Biol. Chem. 278, 32978–32986 (2003). 12815059 10.1074/jbc.M301936200

[b32] Dumitru C. A. & Gulbins E. TRAIL activates acid sphingomyelinase via a redox mechanism and releases ceramide to trigger apoptosis. Oncogene 25, 5612–5625 (2006). 16636669 10.1038/sj.onc.1209568

[b33] Reeves P. J., Callewaert N., Contreras R. & Khorana H. G. Structure and function in rhodopsin: high-level expression of rhodopsin with restricted and homogeneous N-glycosylation by a tetracycline-inducible N-acetylglucosaminyltransferase I-negative HEK293S stable mammalian cell line. Proc. Natl Acad. Sci. USA 99, 13419–13424 (2002). 12370423 10.1073/pnas.212519299 PMC129688

[b34] Otwinowski Z. & Minor W. in Methods in Enzymology Vol. 276, 307–326Academic Press (1997). 10.1016/S0076-6879(97)76066-X 27754618

[b35] Battye T. G., Kontogiannis L., Johnson O., Powell H. R. & Leslie A. G. iMOSFLM: a new graphical interface for diffraction-image processing with MOSFLM. Acta Crystallogr. D Biol. Crystallogr. 67, 271–281 (2011). 21460445 10.1107/S0907444910048675 PMC3069742

[b36] Winn M. D. . Overview of the CCP4 suite and current developments. Acta Crystallogr. D Biol. Crystallogr. 67, 235–242 (2011). 21460441 10.1107/S0907444910045749 PMC3069738

[b37] Kabsch W. XDS. Acta Crystallogr. D Biol. Crystallogr. 66, 125–132 (2010). 20124692 10.1107/S0907444909047337 PMC2815665

[b38] Karplus P. A. & Diederichs K. Linking crystallographic model and data quality. Science 336, 1030–1033 (2012). 22628654 10.1126/science.1218231 PMC3457925

[b39] Terwilliger T. C. . Decision-making in structure solution using Bayesian estimates of map quality: the PHENIX AutoSol wizard. Acta Crystallogr. D Biol. Crystallogr. 65, 582–601 (2009). 19465773 10.1107/S0907444909012098 PMC2685735

[b40] Cowtan K. The Buccaneer software for automated model building. 1. Tracing protein chains. Acta Crystallogr. D Biol. Crystallogr. 62, 1002–1011 (2006). 16929101 10.1107/S0907444906022116

[b41] McCoy A. J. . Phaser crystallographic software. J Appl Crystallogr 40, 658–674 (2007). 19461840 10.1107/S0021889807021206 PMC2483472

[b42] Afonine P. V. . Towards automated crystallographic structure refinement with phenix.refine. Acta Crystallogr. D Biol. Crystallogr. 68, 352–367 (2012). 22505256 10.1107/S0907444912001308 PMC3322595

[b43] Emsley P., Lohkamp B., Scott W. G. & Cowtan K. Features and development of Coot. Acta Crystallogr. D Biol. Crystallogr. 66, 486–501 (2010). 20383002 10.1107/S0907444910007493 PMC2852313

[b44] Schrödinger L. L. C. The PyMOL Molecular Graphics System, Version 1.3r1 (2010).

[b45] Chemical Computing Group Inc. Molecular Operating Environment, Version 2014.09, www.chemcomp.com (2014).

[b46] Li H., Robertson A. D. & Jensen J. H. Very fast empirical prediction and rationalization of protein pKa values. Proteins 61, 704–721 (2005). 16231289 10.1002/prot.20660

[b47] Dolinsky T. J., Nielsen J. E., McCammon J. A. & Baker N. A. PDB2PQR: an automated pipeline for the setup of Poisson-Boltzmann electrostatics calculations. Nucleic Acids Res. 32, W665–W667 (2004). 15215472 10.1093/nar/gkh381 PMC441519

