# Supplementary material for: Human acid sphingomyelinase structures provide insight to molecular basis of Niemann–Pick disease
Source: Nat Commun. 2016 Oct 11;7:13082. doi: 10.1038/ncomms13082 (PMC5062611; doi:10.1038/ncomms13082)
Supplement: Supplementary Information — Supplementary Figures 1-6, Supplementary Table 1 and Supplementary References [file ncomms13082-s1.pdf]

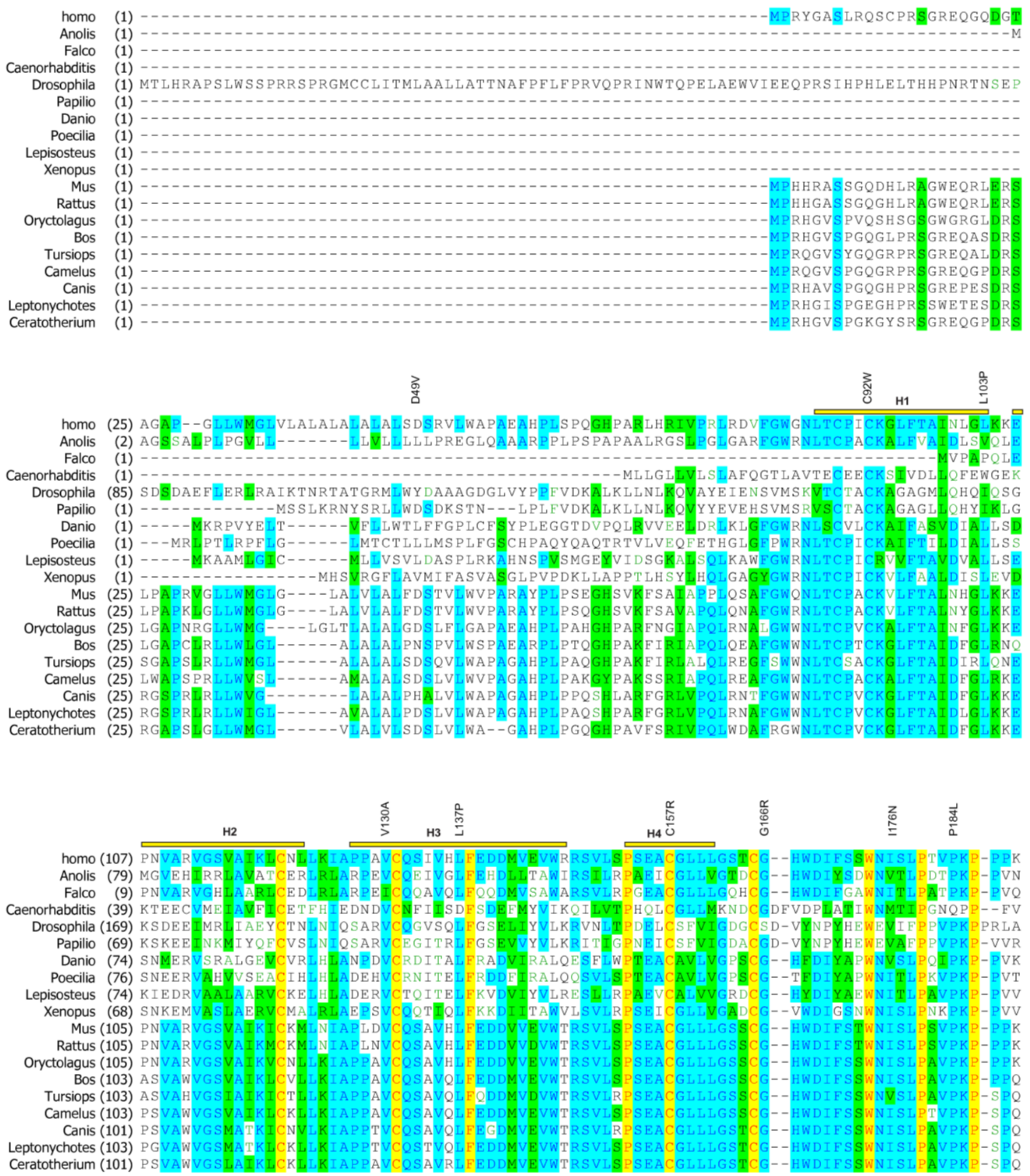

Supplementary Figure 1, page 1 of 3

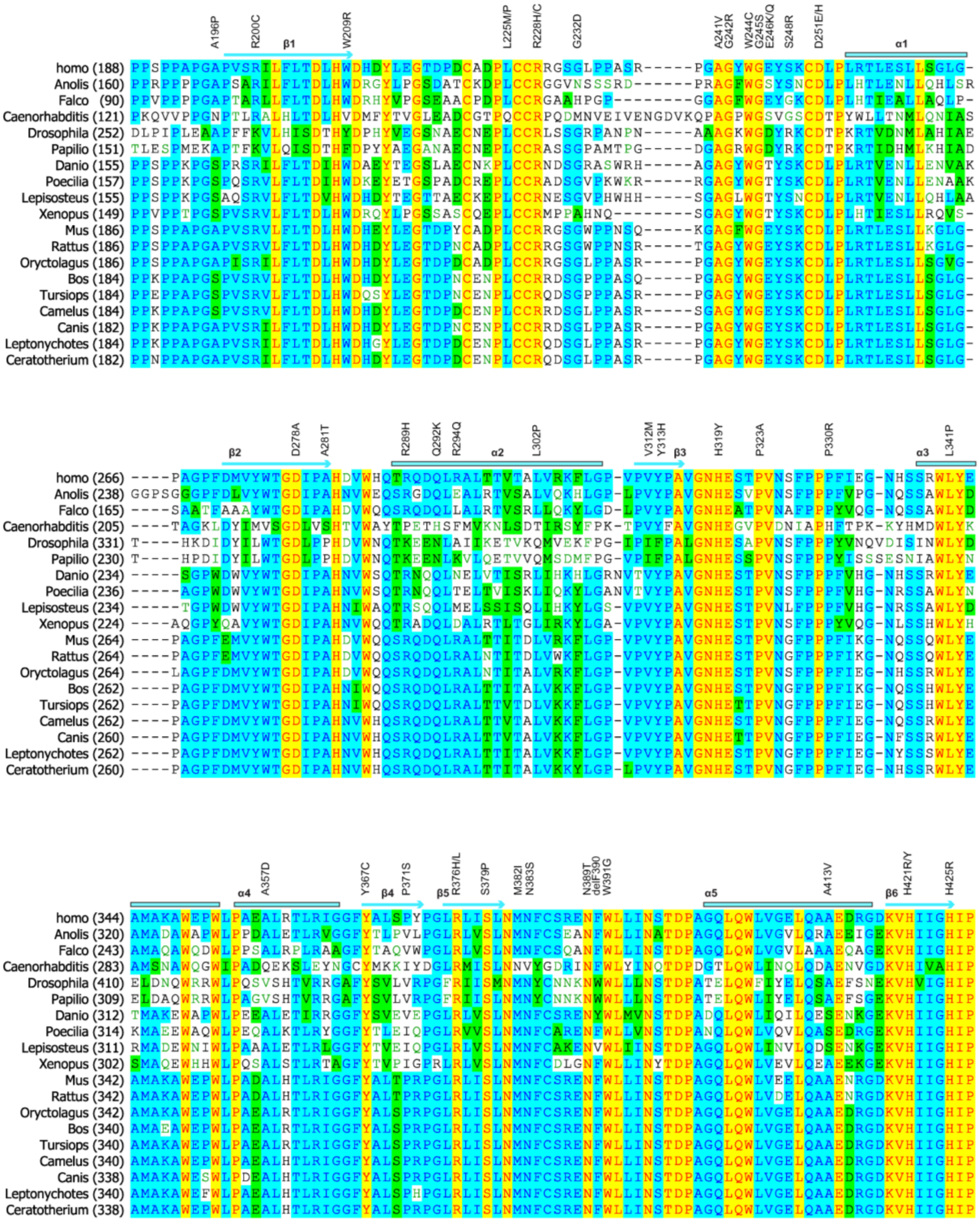

Supplementary Figure 1, page 2 of 3

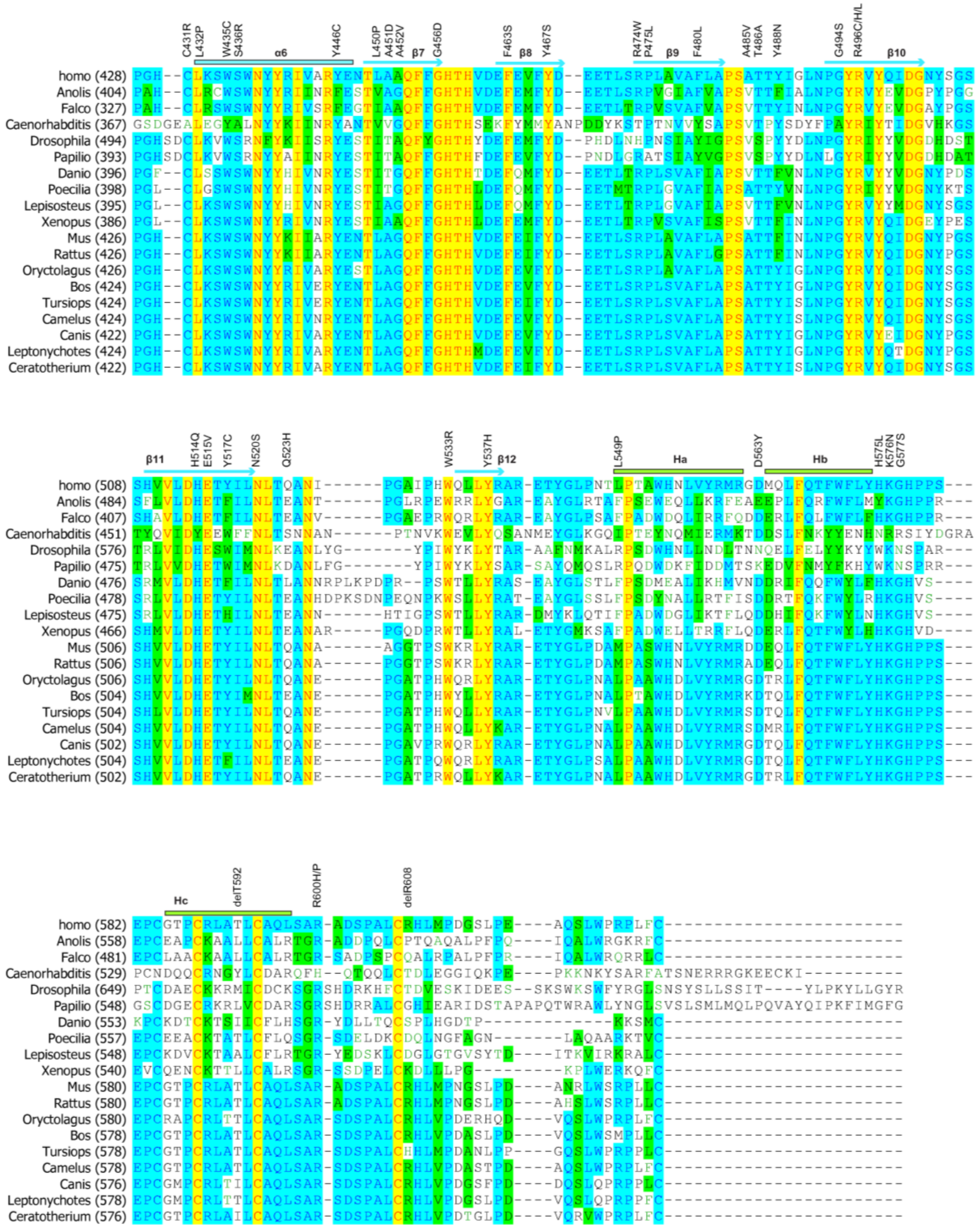

Supplementary Figure 1, page 3 of 3

Supplementary Figure 1. Sequence alignment of ASM from different species. Alignment was performed with Vector NTI and default settings (Invitrogen). Identical residues are in red with yellow highlight. Conserved residues (>50%) are in dark blue with cyan highlight. Similar residues are in black with green highlight. Weakly similar (>20%) residues are in green with no highlight. ASMD mutations

reported in UniProt database are labeled. Human: sp_P17405, or gi:224471897, Canis_lupus: gi_57102910, Camelus_ferus: gi_946650969, Ceratotherium_simum: gi_478488034, Oryctolagus_cuniculus: gi_291384487. Leptonychotes_weddellii: gi_585192661, Tursiops_truncatus: gi_470643762, Bos_taurus: gi_115496992, Rattus_norvegicus: gi_55741778, Mus_musculus: gi_6755582, Anolis_carolinensis: gi_637376492, Xenopus_tropicalis: gi_301628826, Falco_peregrinus: gi_529422001, Lepisosteus_oculatus: gi_573880250, Poecilia_formosa: gi_617356876, Danio_rerio: gi_68367280, Papilio_polytes: gi_389610779, Drosophila_willistoni: gi_195436372, Caenorhabditis_elegans: gi_115532952.

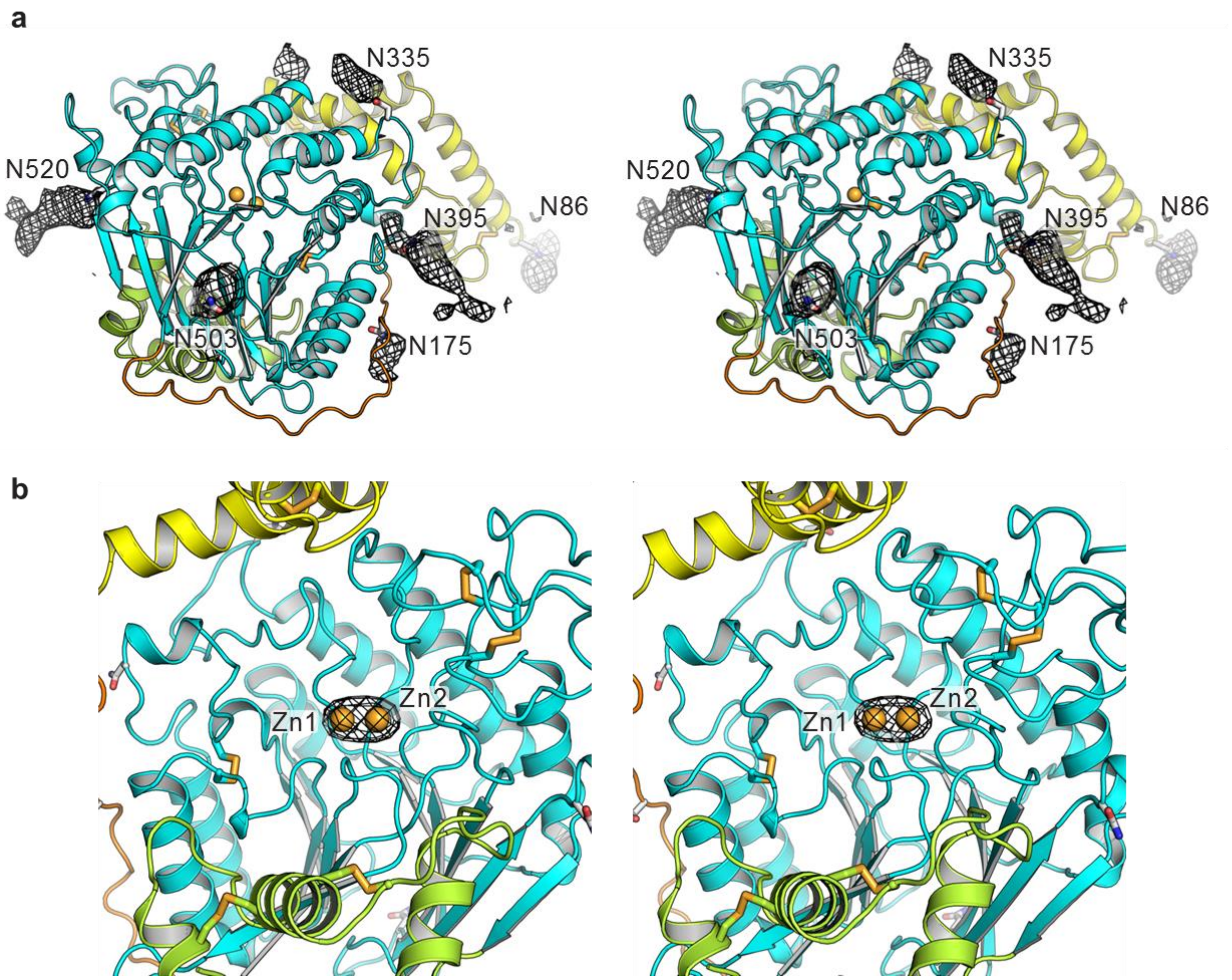

Supplementary Figure 2. Stereo views of electron density observed in olipudase alfa structure. A) 5 glycosylation sites in the catalytic domain, shown as omit Fo-Fc density contoured at 3σ. B) Anomalous difference density for Zn at 8σ.

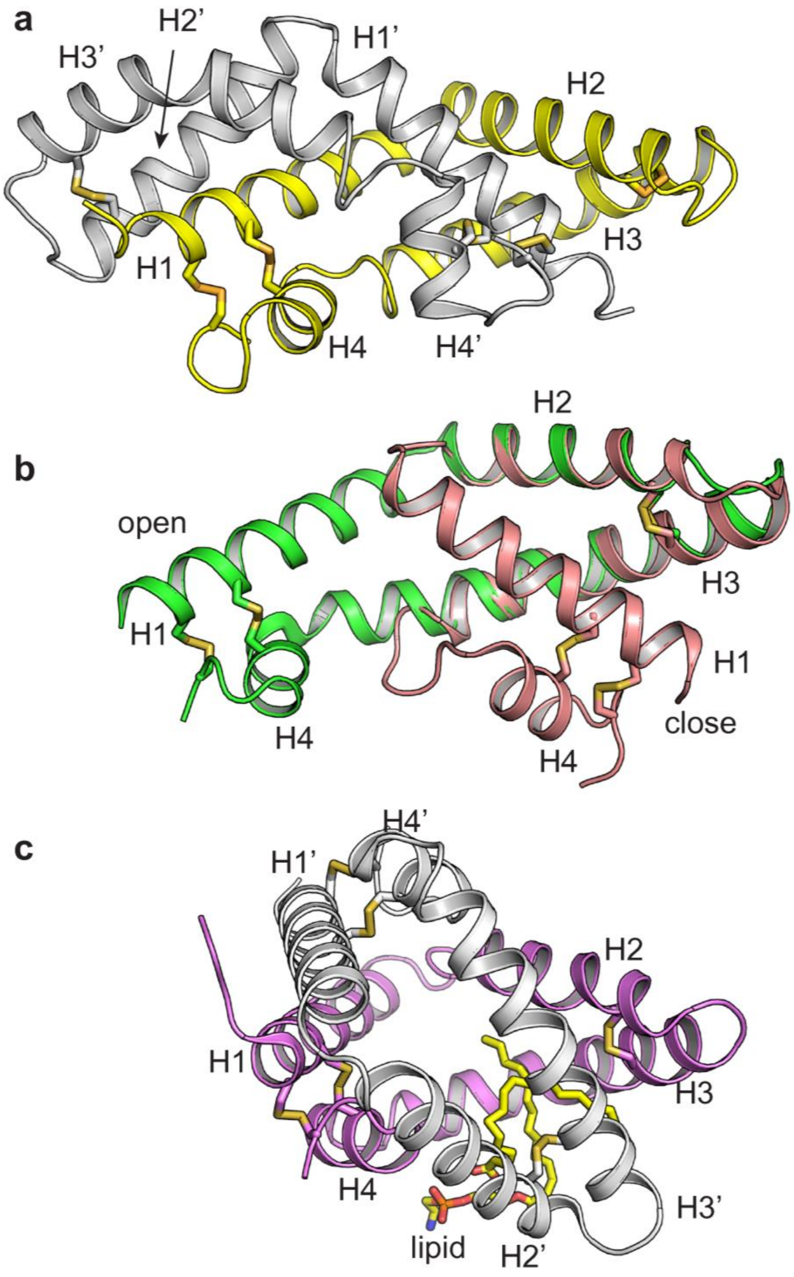

Supplementary Figure 3. Conformations of ASM saposin domain and other saposin proteins. A. Open conformation of saposin and its crystallographic symmetry in ASM. B. Open (green) and closed (salmon) saposin A conformations. The open structure has detergent LDAO bound (PDB ID 4DDJ) [1]. The closed structure has an empty binding site (PDB ID 2DOB) [2]. C. The open conformation of the saposin B dimer in the presence of lipid (PDB ID 1N69) [3]. Lipid is shown as sticks.

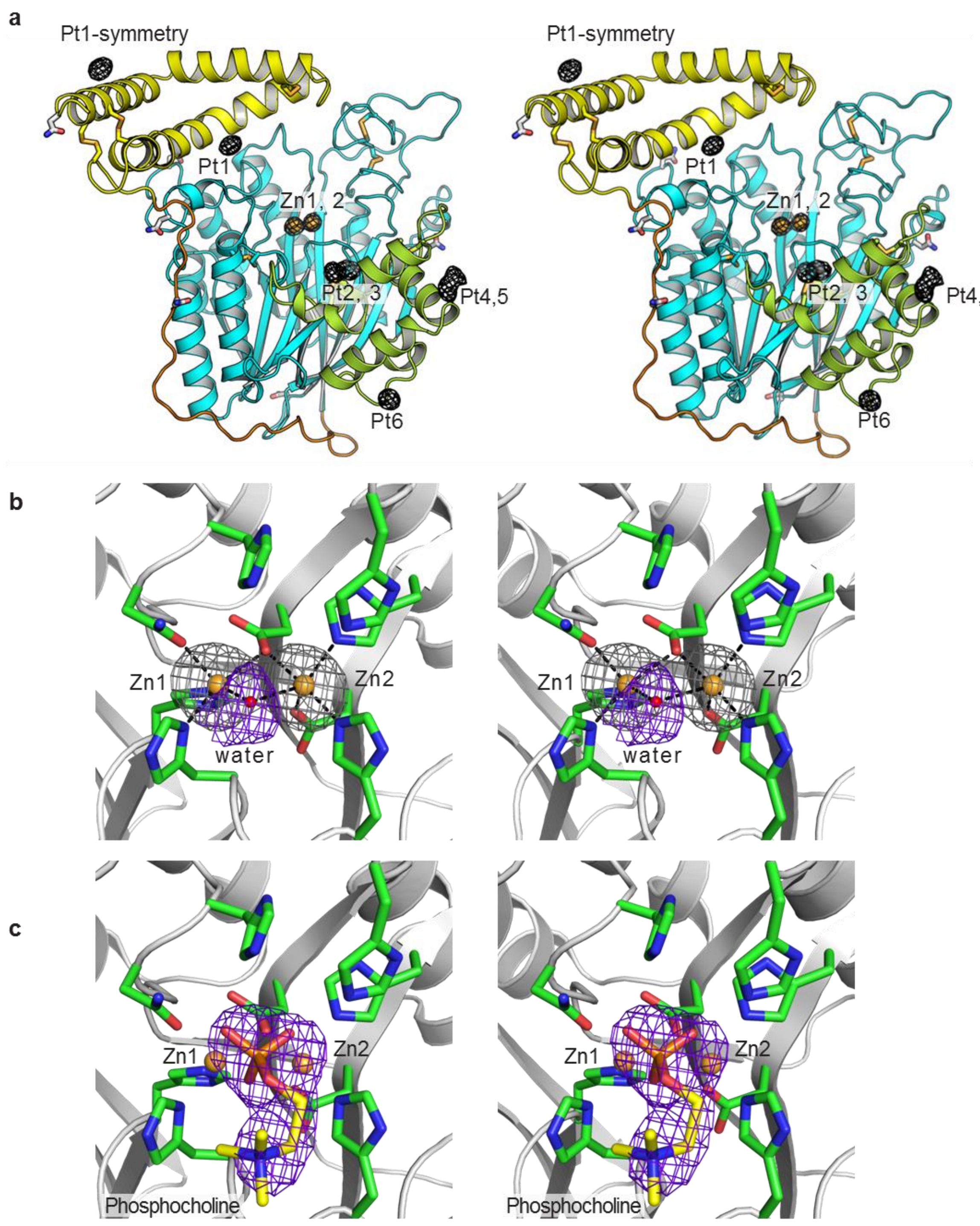

Supplementary Figure 4. Stereo views of electron density of Pt, Zn, water, and phosphocholine. A) Six sites for Pt. Shown are anomalous difference map contoured at 8σ. B) Shown in black is anomalous difference density for Zn at 8σ. Purple density is the omit Fo-Fc map contoured around water at 3σ. C) Omit Fo-Fc density around phosphocholine at 3σ.

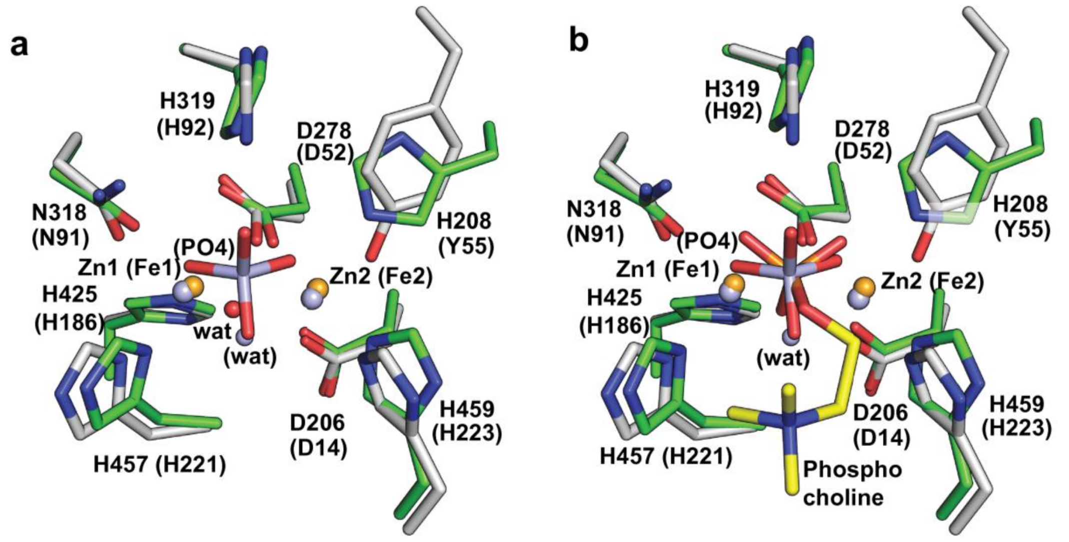

Supplementary Figure 5. Superimposition of active sites of holo ASM (A) and phosphocholine bound (B) to mammalian purple acid phosphatase (PAP, PDB ID 1UTE) [4]. Carbon atoms are green in ASM and grey in PAP. Only Zn binding residues are shown as sticks. PAP compositions are shown and numbered in parentheses next to position equivalent items in ASM.

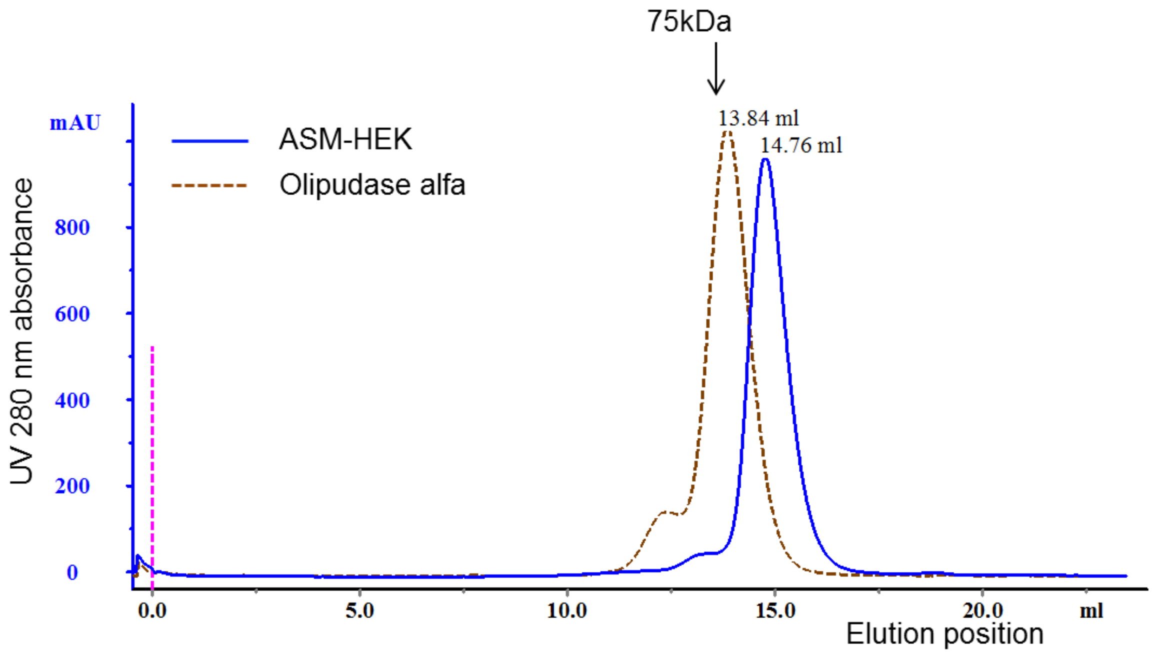

Supplementary Figure 6. Superdex 200 10/300 GL gel-filtration profiles of ASM from HEK293S Gnt1- cells (ASM-HEK) and olipudase alfa.

Supplementary Table 1. Analysis of reported mutations in ASMD patients.

Mutation	NPD[$]	RSA*	Interpretation of mutations based on structure	Category[¶]
D49V	B	NA	NA	NA
C92W	B	0.08	Forms disulfide with C157. Small room between helices for bigger side chain on tryptophan.	Folding
L103P	A, B	0.19	At the end of H1 in saposin, and mutation to proline applies more constraints and decreases the flexibility on the short hinge between H1 and H2.	Folding
V130A	B	0.06	At the N-end of H3. Mediates hydrophobic interactions between saposin and catalytic domain (β1-α1 and β3-α3 loops).	Folding
L137P	B	0.16	In the middle of H3 in saposin. Proline breaks helix.	Folding
C157R	B	0.00	Form disulfide with C92. Breaks disulfide constraint. Small room between helices for big side chain on tryptophan.	Folding
G166R	B	0.17	Adjacent to disulfide C89-C165 in saposin. Small space for bulky side chain on arginine.	Folding
I176N	B	0.09	Mutation introduces hydrophilic group to hydrophobic local environment.	Folding
P184L	A, B	0.31	First P in a PPP sequon in the proline-rich linker. Mutation loosens constraints on the linker and introduces a solvent exposed hydrophobic residue.	Folding
A196P	B	0.25	At the sharp turn between proline-rich linker and β1 in catalytic domain.	Folding
R200C	B	0.24	Guanidine group interacts with α1 and β9.	Folding
W209R	A	0.09	Mutation introduces charges to hydrophobic core. Disrupt β1 folding, thus affect D206, H208 coordination with Zn.	**Active**
L225M	B	0.01	Methionine side chain will clash with His282.	Folding
L225P	B	0.01	Affect constraints on β1-α1 loop.	Folding
R228H	A, B	0.02	Guanidine group form strong interacts with D210 on β1.	Folding
R228C	B	0.02	See R228H. A free thiol may also mess up disulfide formation in local.	Folding
G232D	B	0.44	Solvent exposed, on β1-α1 loop. Could be mild affect to structure/folding.	Folding
A241V	A, B	0.08	Buried side chain in hydrophobic local. Small space for valine.	Folding
G242R	B	0.12	Small space for arginine. Disrupt folding.	Folding
W244C	B	0.13	Side chain interacts with R255, P531, and A524	Folding
G245S	A, B	0.00	Disrupt the tightly folded local environment by W244, Y243 and Y213.	Folding
E246K	A	0.19	Carboxylic acid group interacts with G242 main chain amino N, G240 carbonyl O and Y247 main chain N.	Folding
E246Q	B	0.19	Breaks the interaction between Y247 main chain amino N.	Folding
S248R	A, B	0.13	Hydroxyl oxygen hydrogen bonds to P219 main chain carbonyl O.	Folding
D251E	A, B	0.00	Carboxylic acid group interacts with H282 main chain amino N and its side chain N, and T486 hydroxyl O.	**Active**
D251H	A	0.00	See D251E	**Active**
D278A	A, B	0.00	Carboxylic acid group coordinate binding of both zinc atoms.	**Active**

A281T	B	0.02	Carbonyl O hydrogen bonds to H319 imidazole N. No space for threonine.	**Active**
R289H	B	0.12	Pi-cation interaction with W340, and guanidine group hydrogen bonds to carbonyl O on N335 main chain and side chain and S337.	Folding
Q292K	A, B	0.03	Buried, small room for longer side chain in lysine. Glutamine sidechain is involved in hydrogen bonding network with His319, which is a key residue in catalysis.	**Active**
R294Q	A	0.33	Guanidine group form pi-cation interaction with the imidazole ring on H211 at the end of β1.	Folding
L302P	A	0.12	In the middle of α2 helix. Mediating hydrophobic interactions between α1 and α2 helices. Proline breaks α2.	Folding
V312M	B	0.01	Methionine rotamers cannot fit in the compact hydrophobic environment.	Folding
Y313H	A	0.05	Mutation decreases stability of the hydrophobic environment maintained by the bulky side chain of tyrosine.	Folding
H319Y	A	0.09	Essential imidazole ring for catalysis. Long side chain of tyrosine disrupts catalytic reaction.	**Active**
P323A	B	0.02	At a sharp turn on β3-α3 loop. On interface between saposin and catalytic domain.	Folding
P330R	B	0.33	Last P in a PPP sequon, partially exposed. Mutation breaks hydrophobic contacts to side chain of A400, which covers a hydrophobic pocket.	Folding
L341P	A, B	0.01	Mutation breaks the extensive hydrophobic interactions on leucine side chain and the helix turn.	Folding
A357D	B	0.00	Compact hydrophobic environment.	Folding
Y367C	A	0.00	Extensive hydrophobic contacts. Hydrogen bonds to carbonyl oxygen on P314 on β3.	Folding
P371S	B	0.14	Mutation breaks hydrophobic contacts with L377 and M272.	Folding
R376H	B	0.11	Breaks contacts with E409 on α5, E356 on α4, and amino N on S370 on β4.	Folding
R376L	B	0.11	See R376H	Folding
S379P	B	0.00	In β4 and hydroxyl O hydrogen bonds to carbonyl O on P314.	Folding
M382I	A, B	0.00	Compact hydrophobic environment for long linear side chain on Met. Branched isoleucine cannot fit.	Folding
N383S	B	0.01	Breaks extensive hydrogen bonds on asparagine side chain with G364, Y342, and N383.	Folding
N389T	A	0.01	Breaks extensive hydrogen bonds on asparagine side chain with G325, F327, and N383.	Folding
delF390	A	0.11	In the first turn of a 3_{10} helix in β5-α5 loop. Phenyl ring mediates hydrophobic interactions with neighboring residues.	Folding
W391G	B	0.00	Bulky side chain makes extensive hydrophobic interactions.	Folding
A413V	B	0.00	Buried. Small room for valine.	Folding
H421R	A	0.02	Extensive contacts with neighboring residues. Small room for arginine.	Folding
H421Y	B	0.02	Buried. Small room for tyrosine.	Folding
H425R	B	0.00	Direct coordination with zinc. Mutation disrupts zinc binding and catalytic reaction.	**Active**

C431R	B	0.00	Buried disulfide. Small room for arginine. Disrupt folding.	Folding
L432P	B	0.16	Proline ring will clash with C385, which is disulfide linked to C431.	Folding
W435C	B	0.01	Buried. Makes extensive hydrophobic contacts in local environment. Free cysteine can attack C431-C385 disulfide.	Folding
S436R	B	0.00	Buried. Small room for arginine.	Folding
Y446C	A	0.00	Buried. The bulky side chain makes extensive contacts and stabilizes neighboring secondary structures.	Folding
L450P	A	0.09	In last turn of helix. Proline ring will clash with Y446, and loosens hydrophobic contacts with L443 and I422.	Folding
A451D	B	0.16	Mostly buried. Small room for bulky aspartic acid.	Folding
A452V	B	0.01	Buried. Small room for bulky valine.	Folding
G456D	B	0.00	Buried. Small room for bulky aspartic acid.	**Active**
F463S	A	0.03	Phenyl ring mediates extensive hydrophobic contacts.	Folding
Y467S	A	0.00	Bulky tyrosine side chain mediates extensive contacts. Mutation to serine destabilizes folding.	Folding
R474W	B	0.40	Arginine forms two pairs of salt bridges with E447. Mutation destabilizes folding.	Folding
P475L	A, B	0.13	Small space for leucine. Mutation disrupts folding.	Folding
F480L	B	0.03	In a hydrophobic core. Mutation loses contacts to V478, V511 and W553.	Folding
A482E	A	0.00	Buried. Small room for glutamic acid.	Folding
A485V	B	0.01	Small room for valine. All rotamers introduce clashes in the hydrophobic pocket.	**Active**
T486A	B	0.00	Mutation loses hydrogen bond between T486 hydroxyl O and D251 carboxylic acid group.	**Active**
Y488N	B	0.19	Breaks pi-pi stacking between Y488 and H282, which is essential for catalytic reaction.	**Active**
G494S	B	0.01	Buried. Small space for serine. Mutation disrupts folding.	Folding
R496C	B	0.01	Buried. Arginine is involved in extensive hydrophobic contact (Y496, F203), pi-cation (H514), salt bridge (D461), and hydrogen bonds (T516, Y496).	Folding
R496H	A	0.01	See R496C	Folding
R496L	A	0.01	See R496C	Folding
H514Q	B	0.03	Buried in a hydrophobic pocket. Pi-cation interacts with R496. Mutation to Q introduces a big hydrophilic group.	Folding
E515V	B	0.06	Exposed and involved in charge interactions with R538 and N499. Mutation destabilizes folding.	Folding
Y517C	A	0.10	Bulky side chain covers a hydrophobic pocket, and hydroxyl group is exposed. Mutation opens the hydrophobic pocket.	Folding
N520S	B	0.02	Loses N-glycan.	Folding
Q523H	B	0.45	Exposed and hydrogen bonds to Q534 and glycan on N520. Mutation to histidine causes clashes.	Folding
W533R	B	0.08	Buried in a hydrophobic pocket. Mutation to arginine introduces charged guanidine to the pocket.	Folding
Y537H	A	0.06	Hydroxyl oxygen hydrogen bonds to D461. Phenyl ring is in a	Folding

			hydrophobic pocket involving T516, T542 and L573.	
L549P	B	0.15	First residue on helix Ha in C-terminal domain. Mutation re-orientates the helix.	Folding
D563Y	B	0.38	N-cap on Hb by H-bonding to backbone nitrogens at the Hb C terminus in the C-terminal domain.	Folding
H575L		0.10	His forms close contact to W571 and Y574, which constrain the orientation of helices in C-terminal domain.	Folding
K576N	B	0.00	Buried. Amide on side chain forms 3 hydrogen bonds with main chain carbonyl on Ser484, Thr486, and Asn492.	Folding
G577S	A	0.10	In a hydrophobic pocket covered by N-glycan. Small room for serine. Mutation destabilizes folding.	Folding
delT592	A	0.09	In the middle of Hc helix. A deletion not only breaks the C594-C607 disulfide, but also interferes the interactions between C-terminal domain and catalytic domain. .	Folding
R600H	B	0.07	Extensive contacts: pi-cation (W437), salt bridges (E469), hydrophobic (Y440), hydrogen bond (S603).	Folding
R600P	B	0.07	See R600H	Folding
delR608	B	0.38	In a 3_{10} helix. Deletion of R608 affects the most C-terminal residues 609-629.	Folding

$ NPD: Type A mutation causes early onset neuronopathic phenotype, and type B is non-neuronopathic form.

* GXG ratio: The ratio (x100) of the residue surface area (RSA) to that of the corresponding GXG tripeptide. Calculated with areaimol in CCP4 [5].

¶ Categorization is prediction based on structures.

Supplementary References

1. Popovic, K., Holyoake, J., Pomes, R. & Prive, G.G. Structure of saposin A lipoprotein discs. *Proc Natl Acad Sci U S A* **109**, 2908-12 (2012).
2. Ahn, V.E., Leyko, P., Alattia, J.R., Chen, L. & Prive, G.G. Crystal structures of saposins A and C. *Protein Sci* **15**, 1849-57 (2006).
3. Ahn, V.E., Faull, K.F., Whitelegge, J.P., Fluharty, A.L. & Prive, G.G. Crystal structure of saposin B reveals a dimeric shell for lipid binding. *Proc Natl Acad Sci U S A* **100**, 38-43 (2003).
4. Guddat, L.W. et al. Crystal structure of mammalian purple acid phosphatase. *Structure* **7**, 757-67 (1999).
5. Winn, M.D. et al. Overview of the CCP4 suite and current developments. *Acta Crystallogr D Biol Crystallogr* **67**, 235-42 (2011).
